# Statistics and Medicine: the Indispensable Know-How of the Researcher

**Published:** 2013-01-04

**Authors:** R Romano, E Gambale

**Affiliations:** 1 Department of Medicine, University of Salerno; 2 Department of UTTP-FOS, ENEA

**Keywords:** Statistics, Experimental and Observational Studies, Parametric and Nonparametric-Parametric Methods, P Value

## Abstract

Statistics has often been misunderstood in Medicine, but it is indispensable knowledge both for the experimenter and the reader. Statistical methods allow to study diseases, patients, and epidemiological events. The modern researcher cannot refuse to know and to use statistics. A deeper understanding is required to prepare a research project and to avoid colossal mistakes of misleading.

The aim of this paper is to provide an organized and structured point of view on the use of statistics in Medicine and Research, showing the principal resources to organize a scientific study from the declaration of a hypothesis to the report of the results.

“Statistical thinking will one day be as necessary for efficient citizenship as the ability to read or write.”

*H. G. Wells*

## I. INTRODUCTION

Statistics represents the heel of Achilles for the modern researcher. The necessity to apply statistics to any scientific problem leads the researcher to assume the burden of a deeper knowledge in this science.

Medicine often uses probabilistic statistics that could be far away from the scientific method. Data are used and analysed in order to highlight trends or to make a prevision for the validity of a diagnostic method, a therapy or a prognosis for a disease. When an event is pointed out in a large amount of cases, a descriptive method is used to identify the potential basic mechanism. Starting from this point, the aim of the researcher should be the precise and reasoned planning of a scientific project. Any aspect of a research project should be provided at the beginning and then verified.

The data from the study of a subset of the population, the sample, are collected and then subjected to statistical analysis, in order to attend to two purposes: description and inference.

Descriptive statistics uses data to describe numerically and graphically the observations in the sample. Instead, inferential statistics uses the identification of patterns in data to draw inferences about the population.

Statistical analysis might identify the presence of a relationship between variables.

The need for greater knowledge in statistics is crucial both for the researcher, who is planning a study or elaborating data from a study, and for readers to understand the plan of a study and the validity of its conclusion.

## II. STATISTICAL METHODS

### A) Experimental and observational studies

The use of statistical studies allows to evaluate the causality and to draw a conclusion about the effect of an independent variable on the dependent variables examined. We consider two types of statistical study: experimental and observational (Table 1). They differ in how studies are conducted and in their aims [1,2].

An observational study evaluates the inferences about the effect of a treatment on patients, when the option of giving a patient to a treated group versus a control group is not an option for the investigator. Data are already available, researchers can explore any similarities among them. So, it is characterized by the observation of the interaction between two or more variables: the independent variable (or the risk factor) and, on the other hand, the dependent variables (or the response). This type of study might be prospective (with the evaluation of the relative risk) or retro-prospective (with the evaluation of the odds ratio). It is essential to consider the presence of confounding variables that influenced the relation between the risk factor and the response causing a bias. This study might be preliminary to an experimental study, the starting point to plan a research.

The experimental model is fundamental for the scientific model: after the observation of a phenomenon, the hypothesis is declared and then verified. Nowadays, planning a research project and identifying a hypothesis clearly, it is necessary to check its innovation. Only after that the researcher can design and perform the experiment and at the end evaluate the validity of the hypothesis.

The substantial difference between the observational and experimental study is in the role of the researcher. In an experimental study researchers are involved in taking measurements, manipulating the system, and then taking again measurements using the same procedure in order to understand the impact of the manipulation on the system. Instead, in an observational study there is no experimental manipulation but only the data collection and their analysis.

Designing an experimental study, the researcher needs to:

- consider the initial information about the entity of the effects, the alternative hypothesis and the experimental variability;
- plan the research and design experiments, choosing the quantity to measure, the independent and the dependent variables, the method of measurement, the acceptable error of measure, the variability range, the acceptable influence of confounding variables;
- perform the experiment and measurement;
- analyse data following strict determined before;
- present the results of the study.

Planning a study the researcher decides to support an “hypothesis”, a conjecture about the variables taken into account in a population. This hypothesis can be verified with statistical method, e.g. confirmatory tests and χ^2^ test.

### B) The choice between parametric and non-parametric methods

A parametric model is a distribution that can be described using a finite number of parameters. Many basic statistical methods are parametric. The use of parametric methods leads to make more assumptions than using non-parametric methods. If these assumptions are correct, parametric methods can be accurate and precise.

The necessity to make assumptions is a risk, because if they are wrong, the parametric method will be misleading. This is the reason why they are considered statistical powerful, but not robust.

In addition to parametric models, there are also semi-parametric, semi-nonparametric, and non-parametric models. A non-parametric model is a model where all parameters are in infinite-dimensional parameter spaces. It considers data belonging to any particular distribution or techniques.

The use of non-parametric methods may be indispensable when data are characterized by a ranking without an useful numerical interpretation. Non-parametric methods make fewer assumptions than parametric methods. So they can be applied widely and be considered robust. Nonparametric tests have less power where a parametric test would be appropriate [3].

### C) The evaluation and control of the error

The choice of a sample might be extremely difficult. The sample should be representative of an entire population to be used as a guide. The use of a representative sample assures that the inferences and conclusions can be safely extended from the sample to all the population. A crucial problem is in determining the adequate size of the sample. The sample should be large enough to be representative, but it does not need to be oversize to reduce the costs of the study.

There are two large groups of errors:

- the error due to measurement instrumentation and to the operator (for example, systematic error, sensitivity error). It is a controllable but often unavoidable error, due to a discretional evaluation of the measurement. In the medical field, much emphasis is given to error “bias” is an error of this kind due to experimenter’s choices.
- the statistical error, that is always present when there is a measurement, has many causes, even inherent to the experiment itself. This error can be reduced increasing the number of measurements, but it cannot be eliminated. It is the measure of the amplitude of data sheet dispersion. The normal distribution is frequently used to describe random variables to real value. It is considered the most prominent distribution in statistics because it is a distribution that can be used for a large number of random variables. It is commonly used in practice as a simple model for complex phenomena both in natural science and in social science. The error in an experiment is usually assumed to follow a normal distribution. This assumption is used to calculate the propagation of uncertainty. When the distribution is a Gaussian distribution the error α can be calculate as (1)
  (1) $$\Delta x=\sqrt{\frac{\sum {\left(x_{i}-\mu \right)}^{2}}{\left(n-1\right)}}$$
  where *n* is the sample size and μ is the mean of the distribution [3].

Many of the errors derived from the measurements are classified as “noise” if they are random. They can lead to statistic misleading. Misuse of statistics can produce subtle, but serious errors in description and interpretation. Even experienced professionals make such errors, but they can lead to enormous decision errors.

### D) Significance levels

Statistics offers some instruments to understand if the results of the measurements is that expected by the hypothesis. One of these is the significance test.

A significance test evaluates the plausibility of the observed data when a “null hypothesis” is true. It is expressed by a significance level α that is the probability of rejecting a null hypothesis that is true [3].

In the interpretation of the statistical information, it is possible to evaluate the null hypothesis wrongly.

As reported in Table 2, there are two types of error:

- Type I error or α, when the null hypothesis is rejected, but it is true. It is a “false positive”.
- Type II error or β, when the null hypothesis is false, but it is not rejected. It is a “false negative”.

Rejecting a true null hypothesis is an error that should be as small as possible. It is necessary to choose the smallest value: 0.01, 0.05 or 0.001. This significance level is named P value. It is used to express the chance that the observed data are due to chance. The relevance of the chosen P value might depend on the sample size. Furthermore, reporting results it is important to report the real P value.

It is difficult to choose a significance level, but it is extremely difficult interpreting results using the P value. If P value of observed data is small enough (P< 0.05 or P< 0.01 or P< 0.001), the null hypothesis could be rejected, and the alternative hypothesis could be accepted. But if P value is not small enough, there is no statistical significance. The null hypothesis cannot be rejected, but it does not mean anything else.

### E) Survival statistics

Survival statistics is a branch of statistics that deals with death in a biological organism. Time is a fundamental character of this branch and the main “events” are death and failure. Survival models can be usefully considered as ordinary regression models in which time is the response variable.

Among the estimators used to estimating survival data, the Kaplan–Meier estimator is extremely important. In the medical literature, it is frequently used to measure the rate of patients living after a therapy or after the exposition to a risk factor. A plot of the Kaplan–Meier estimate of patients’ survival is made by horizontal steps of declining magnitude. The value of the survival function is constant between clicks that are successive distinct sampled observations.

Among survival analysis, it is necessary to consider the survival rate. It is percentage of people alive after 1, 5 or 10 years after the diagnosis of disease. It can be expressed as relative survival, cause specific survival, disease specific survival. The interval considered can be one, five, and ten years. Survival rates can be used to compare the effectiveness of treatments and to evaluated the prognosis of patients [1].

## III. REPORTING STATISTICAL RESULTS

Planning and reporting an observational or an experimental study it is important to declare the aim of the study, the statistical method that will be used and the endpoints. The characteristics of subjects and of the planning should be clearly expressed. It is important if the study is blinding (masking). The endpoints are the events that will be considered. It is possible to differentiate primary and secondary endpoints. Examples of endpoints can be death (in a study survival rate), adverse events and morbidity, toxicity, maximum tolerated dose, response to a therapy. Every adverse event should be reported.

The software used for analysing data should be reported, e.g. SPSS or SAS. So, statistical tests should be described. There should be the type of statistical test, if it is a confirmatory analysis or if it is an exploratory test that need to be strengthen with cross validation [1, 4].

Criteria of inclusion or exclusion are indispensable to clarify the validity of interpretation of the data and the extent to which the results can be applied.

Data should be enlighten and so their sources [4].

Reporting results, any exclusion during analysis should be expressed. Data analysis should be reported with appropriate confidence intervals or P value [3,4].

The interpretation of the P value is disputable. The presence of a P value less than a predefined value determines statistical significance, but its absence is not a reason to reject the alternative hypothesis. Statistical significance does not mean equivalence of treatment or techniques because the clinical importance of a result depends on several variables. Careful statisticians distinguish between statistical and medical significance.

To avoid problems in the interpretation of analysis, it is necessary to choose easy endpoints, to avoid using the term significant, to use correction for data when it is due, e.g. the correction of Bonferroni.

Results and data should be reported in a clear and not confused way, using tables and graphs. The estimation of a valuable and important relation among data is facilitated using graphs.

Graphs give a visual perception of results and catch attention quickly. Understanding of the context is immediate if a graph is well-organized. A graph has two component: content and format. The content is what is reported in a graph while the format is the way that it is reported. It is fundamental to avoid the overlapping of data symbols. If the graph requires the use of many lines, choose patterns that are distinguished easily from each other [5].

If visualizing distributions is essential, show the distribution of data with scatter plots or one-way plots, but avoid graphs showing summaries of data such as bar graphs showing means with standard deviation. Good work is a work well reported.

## Figures and Tables

**Table 1:** **Classification of observational and experimental studies.**

CLASSIFICATION OF EPIDEMIOLOGIC STUDIES	
Observational Studies	Cohort
	Cross-sectional
	Case- Control
	Ecological
Experimental Studies	Preventing Trial
	Clinical Trial

**Table 2:** **The null hypothesis and the possible errors.**

	H_0_*true*	H_0_ false
H_0_ non rejected	True positive	Error β
H_0_ rejected	Error α	True negative
